# Dynamic cerebral autoregulation is preserved during isometric handgrip and head‐down tilt in healthy volunteers

**DOI:** 10.14814/phy2.13656

**Published:** 2018-03-29

**Authors:** Maria Skytioti, Signe Søvik, Maja Elstad

**Affiliations:** ^1^ Division of Physiology Institute of Basic Medical Sciences University of Oslo Oslo Norway; ^2^ Institute of Clinical Medicine Faculty of Medicine University of Oslo Oslo Norway; ^3^ Department of Anaesthesia and Intensive Care Akershus University Hospital Lørenskog Norway

**Keywords:** Dynamic cerebral autoregulation, head‐down tilt, isometric handgrip, wavelet analysis

## Abstract

In healthy humans, cerebral blood flow (CBF) is autoregulated against changes in arterial blood pressure. Spontaneous fluctuations in mean arterial pressure (MAP) and CBF can be used to assess cerebral autoregulation. We hypothesized that dynamic cerebral autoregulation is affected by changes in autonomic activity, MAP, and cardiac output (CO) induced by handgrip (HG), head‐down tilt (HDT), and their combination. In thirteen healthy volunteers, we recorded blood velocity by ultrasound in the internal carotid artery (ICA), HR, MAP and CO‐estimates from continuous finger blood pressure, and end‐tidal CO_2_. Instantaneous ICA beat volume (ICABV, mL) and ICA blood flow (ICABF, mL/min) were calculated. Wavelet synchronization index *γ* (0–1) was calculated for the pairs: MAP–ICABF, CO–ICABF and HR–ICABV in the low (0.05–0.15 Hz; LF) and high (0.15–0.4 Hz; HF) frequency bands. ICABF did not change between experimental states. MAP and CO were increased during HG (+16% and +15%, respectively, *P* < 0.001) and during HDT + HG (+12% and +23%, respectively, *P* < 0.001). In the LF interval, median *γ* for the MAP–ICABF pair (baseline: 0.23 [0.12–0.28]) and the CO–ICABF pair (baseline: 0.22 [0.15–0.28]) did not change with HG, HDT, or their combination. High *γ* was observed for the HR–ICABV pair at the respiratory frequency, the oscillations in these variables being in inverse phase. The unaltered ICABF and the low synchronization between MAP and ICABF in the LF interval suggest intact dynamic cerebral autoregulation during HG, HDT, and their combination.

## Introduction

Cerebral blood flow (CBF) is tightly regulated by several mechanisms to ensure neuronal oxygenation in spite of central hemodynamic variability. Cerebral autoregulation (CA) is a key mechanism that ensures a stable CBF over a wide range of arterial blood pressure changes (60–150 mmHg) (Lassen 1959), albeit with great between‐subject variability. Due to its clinical importance, several methods to quantify CA have been proposed. Static CA is the quantification of the steady‐state change in CBF in relation to steady‐state changes in arterial blood pressure. Dynamic CA characterizes the CBF response to abrupt changes in arterial blood pressure or the interplay between spontaneous arterial blood pressure oscillations and CBF velocity oscillations (Aaslid et al. 1989; Tiecks et al. 1995; Zhang et al. 1998). All the metrics of CA proposed however have the same objective: to assess the integrity of CA.

Frequency domain analysis is quite popular in assessing CA (Claassen et al. 2016). Abrupt changes in arterial blood pressure (such as those induced by lower body negative pressure or thigh‐cuff release) cause a simultaneous change in CBF but also elicit an autoregulatory response, within seconds in healthy individuals. Czosnyka et al. (2009) suggest that a delayed CBF response to abrupt changes in arterial blood pressure may indicate an impaired CA. Rapid oscillations in arterial blood pressure induced by oscillatory lower body negative pressure are transmitted to CBF velocity (Tan 2012); hence dynamic CA is considered to operate best in frequencies below 0.15 Hz (Panerai 2008).

Analysis of the spontaneous variability in arterial blood pressure and CBF velocity with wavelet transforms has been proposed as a method to assess the dynamic relationship between arterial blood pressure and CBF (Latka et al. 2005; Peng et al. 2010; Tian et al. 2016). In contrast to transfer function analysis, wavelet analysis can address the nonlinear, nonstationary time‐scaled nature of CA (Panerai 2014; Addison 2015) and could be a method of continuous CA monitoring. The synchronization index *γ* (Latka et al. 2005), varying from 0 to 1, has been used to study the phase difference variability between cardiovascular and cerebrovascular variables. Low values of *γ* (i.e., <0.3) indicate low synchronization between the signals’ oscillations, while high values of *γ* (i.e., >0.6) indicate high interdependence between the two signals (Latka et al. 2005; Peng et al. 2010).

Arterial partial pressure of CO_2_ is a strong regulator of CBF (Claassen et al. 2007; Peebles et al. 2007; Sato et al. 2012; Brothers et al. 2014), and variations in end‐tidal CO_2_ (ETCO_2_) significantly contribute to very low frequency (<0.04 Hz) CBF variability (Mitsis et al. 2004; Peng et al. 2010). In parallel with cardiac output (CO) reductions induced by lower body negative pressure, CBF has been found to decrease, despite unaltered mean arterial blood pressure (MAP) (Ogoh et al. 2005; Skytioti et al. 2016). The effects of dynamic changes in CO on CBF variability are however not well documented. For instance, Deegan et al. (2010) found no correlation between cardiac index and dynamic CA quantified by the autoregulatory index following thigh‐cuff release. Changes in sympathetic or parasympathetic activity may further modify the ability of the cerebral vasculature to regulate against arterial blood pressure changes (Hamner and Tan 2014; Tan and Taylor 2014). Alterations in dynamic CA was found following ganglion blockade, demonstrating that autonomic neural activity affects short term CBF variability (Zhang et al. 2002). Cooke et al. (2003) reported an unaltered dynamic CA following a slight head‐down tilt (HDT) as a nonpharmacological way to decrease sympathetic activity. Acute HDT to −10° resulted in central blood volume increase and sympathetic withdrawal due to arterial baroreceptor loading (Cooke et al. 2003).

Exercise modifies autonomic neural activity due to sympathetic stimulation via the exercise pressor reflex (Wallin and Charkoudian 2007; Secher and Amann 2012). Isometric handgrip has significant effects on the cardiovascular system, that is, an increase in arterial pressure, heart rate (HR) and CO (Elstad et al. 2009). The effects of exercise‐modified cardiovascular oscillations on cerebral circulation are however unclear. Previous studies showed that dynamic CA is preserved during submaximal static handgrip despite an increase in CBF velocity (Ogoh et al. 2010). Other studies report an increase in middle cerebral artery blood velocity (Vianna et al. 2012) and altered dynamic CA (Nogueira et al. 2013) during sympathetic stimulation induced by isometric handgrip.

We aimed to study the relationship between spontaneous variability in central cardiovascular variables (MAP, CO, HR) and CBF in two frequency intervals (0.05–0.15 Hz and 0.15–0.4 Hz). The low frequency interval was used to study dynamic CA and sympathetic influences on circulation, whereas the high frequency interval was used to study respiratory cardiovascular fluctuations. We studied the response of CBF to isometric handgrip in the horizontal supine position and during HDT. In addition, we examined whether CA changes during isometric handgrip both in the horizontal supine position and during HDT. Our hypothesis was that these physiological maneuvers (HDT and HG), which modify autonomic activity and induce cardiovascular changes, would affect cerebrovascular variability and dynamic CA.

## Materials and Methods

### Subjects

Thirteen young healthy volunteers, four males and nine females, median age 22 (range 19–27 years), median body mass index 22 (range 20.4–24.0) were recruited and gave written, informed consent to participate. All procedures conformed to the Declaration of Helsinki. The Regional research ethics committee (ref.no: 2014/2227, January 2015) approved the protocol and procedures.

None of the subjects were tobacco smokers or taking any medication. They were instructed to abstain from caffeine and strenuous physical activity for at least 12 h and alcohol for 24 h before each experiment.

### Experimental protocol

Experiments took place between 11.00 am and 3.00 pm in a quiet room with an ambient temperature of 22–24°C, continuously recorded to ensure stable conditions. All subjects visited the laboratory before the actual experiment to familiarize themselves with the equipment, to have their maximal voluntary handgrip contraction (MVC) determined, and to train keeping a steady handgrip on the dynamometer at their 30% MVC.

Before the recordings, the diameter of the subject's right internal carotid artery (ICA) was obtained at the measurement site using B‐mode Ultrasound (10 MHz and 2.5 MHz, System Five, GE Vingmed Sound, Norway). The subjects lay supine on a tilt bed throughout the procedure, with the neck in the neutral position, that is, no pillow supporting the head. The right arm was comfortably placed on an arm pad. The palm was in close proximity with the dynamometer so that the subjects could start the handgrip maneuver immediately on command.

Recordings were obtained with subjects in the horizontal supine position and during a −10° HDT. After a 3‐min baseline period of rest, the subjects were instructed to perform isometric handgrip at 30% of their MVC for 3 min, continuously getting feedback from the dynamometer. A 5‐min period of rest followed before changing the tilt of the bed and repeating the handgrip procedure. The protocol was run twice for each subject, with a 5‐min pause between rounds. The first round was randomized to start in either horizontal supine position or HDT; in the second round this sequence was reversed.

### Recordings

Mean ICA velocity was recorded continuously by a trained operator (handheld probe), using pulsed Doppler ultrasound (5 MHz probe, insonation angle: 45°, SD‐100, Vingmed, Horten, Norway). ICA velocity was measured approximately 2 cm above the bifurcation of the common carotid artery in order to avoid turbulent flow (Willie et al. 2012). Finger arterial pressure was recorded continuously from the middle left finger (Finometer, Finapres Medical System, Netherlands), the hand being positioned at heart level. Beat‐by‐beat MAP was calculated by numerical integration. The Finometer also provided pulse rate and cardiac stroke volume estimates from the blood pressure curve (SV_bpc_). Respiratory frequency (RF) was recorded using an elastic belt around the abdomen (Respiration and body position amplifier, Scan‐Med a/s, Norway). Instantaneus HR was calculated from the R‐R distance in a three‐lead ECG. Expiratory ETCO_2_ was sampled continuously from near the nares, recorded by sidestream capnography (Cap10, Medlab GmbH, Germany). Blood velocity waves, signals from the respiration belt, ECG, arterial blood pressure waves, pulse rate (Finometer), SV_bpc_ (Finometer), gripping force, and room temperature were sampled at 100 Hz and transferred on‐line to a recording computer running a dedicated data collection and analysis program (Program for real‐time data acquisition, Morten Eriksen, Norway).

ICA beat volume (ICABV, mL/heart beat) was calculated beat‐by‐beat from blood velocity and the diameter of the ICA. ICA blood flow (ICABF, mL/min) and CO estimates (CO_bpc_) were calculated beat‐by‐beat from ICA‐BV and SV_bpc_ (Finometer) respectively, multiplied by instantaneous HR.

### Signal processing and statistical analysis

All datasets were resampled at 4 Hz and analyzed by a custom‐made program written in MATLAB (version R2015b, Mathworks, Natick, MA). For data that were sampled at a higher frequency, linear interpolation was used for the resampling. For data that were sampled beat by beat, values were kept constant for most of the time between beats and then linearly interpolated to the next value. For steady‐state analyses, the recorded signals were inspected and 2‐min periods of continuous, technically successful recordings from each experimental time interval were extracted. All technically successful recordings from each subject were included in the steady‐state analysis. Comparison of the cardiovascular values were made in four different states: Horizontal supine rest (Rest), horizontal supine handgrip (HG), HDT‐rest (HDT), and HDT + HG. Medians and 95% CI over the 2‐min intervals were calculated by Hodges‐Lehmann's estimate (Hollander and Wolfe 1999). The Wilcoxon matched‐pairs signed‐rank test against a two‐sided alternative (Hollander and Wolfe 1999) was used to test differences between states. Friedman test for k related samples was used to test the difference between the first, the second and the third minute of the HG maneuver. (StatXact, Cytel Studio 10, Cytel Inc.). Significance level was set before analyses at *P* = 0.01. Numbers are medians with 95% CI if not otherwise stated.

### Wavelet analysis

The recorded time‐series were also analyzed in the frequency domain, using wavelet transformation. One subject had to be excluded from this analysis because of frequent extrasystoles. The wavelet transform technique, a time‐frequency method with logarithmic frequency resolution, was used to analyze the variability and the interrelation of cardiovascular and cerebrovascular variables. The time‐frequency analysis tools employed in the study are implemented in MATLAB (version R2015b, Mathworks, Natick, MA) and were developed by the Department of Physics, Lancaster university, UK (Iatsenko et al. 2015). The Morlet mother wavelet (Morlet 1983; Bracic and Stefanovska 1998) was selected for the wavelet transform analysis of the cardiovascular variables at four distinct time intervals of 120 s each: Rest, HG, HDT, HDT + HG.

For each time interval, the time‐averaged wavelet spectral power, phase angle and phase coherence were computed in the low‐frequency (LF) interval (0.05–0.15 Hz) and in the high‐frequency (HF) interval (0.15–0.4 Hz). For each of the aforementioned time and frequency intervals, the integral (area under the curve) of the wavelet spectral power was numerically calculated using Simpson's rule. The integrals were used as measures of variability in the frequency intervals.

Interaction between variables was studied by computing time‐averaged phase angles, phase coherence and the phase synchronization index *γ* (Latka et al. 2005). Wavelet phase coherence is well‐correlated with the index *γ*, as by definition the former is the square root of the latter (Addison 2015). We examined the interaction in the following pairs of variables: MAP–ICABF (to study dynamic CA), CO_bpc_–ICABF (to study the effect of CO_bpc_ oscillations on ICABF) and HR–ICABV (to study the regulatory role of HR variability on CBF). The median value of the synchronization index was obtained across each frequency interval. The frequency‐averaged wavelet coherence for the system MAP–ICABF was calculated for each time point from the contour plots of the time‐localized coherence during HG and HDT + HG, in the LF and HF frequency interval. The averaged curve for all subjects was then plotted against time to examine if the relationship between MAP and ICABF changed during the ongoing isometric exercise. In addition, wavelet phase, coherence and *γ* were extracted at each subject's Mayer wave peak (i.e., the peak in the plot of *γ* over frequencies around 0.1 Hz) and at the peak frequency of the signal from each subject's respiratory band, as a substitute for respiratory frequency (RF). The circular mean and 95% CI were calculated for the phase angles using MATLAB toolbox for circular statistics (Fisher 1995; Berens 2009). Two oscillating variables were considered to be in phase when their absolute phase difference was less than 0.79 rad (45°) and in inverse phase when the absolute phase difference was more than 2.35 rad (135°).

## Results

**Figure 1:**
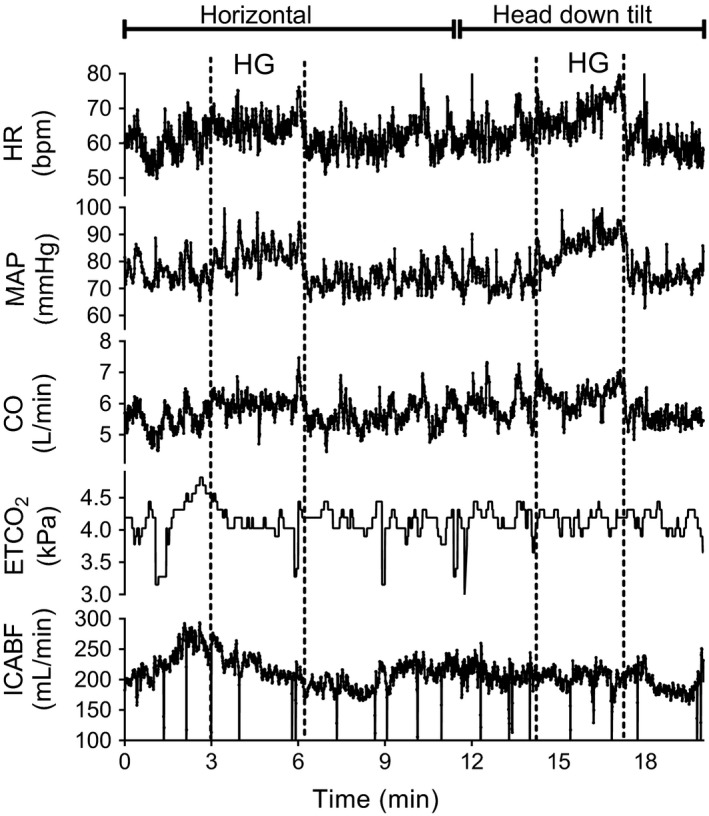
Raw recordings of heart rate (HR), mean arterial pressure (MAP), cardiac output estimates (CO_bpc_), end‐tidal CO_2_ (ETCO_2_) and internal carotid artery blood flow (ICABF) from one representative subject. During isometric handgrip (HG), HR, MAP and CO_bpc_ increased where as ETCO_2_ and ICABF did not change both in horizontal position and during head‐down tilt.

All subjects completed the experimental protocol successfully. Steady‐state cardiovascular and respiratory values in the four experimental states are presented in Table 1. Cardiovascular and cerebrovascular variability is shown in Table 2 (LF interval) and Table 3 (HF interval). Phase angles, coherences and synchronization index *γ* are shown in Table 4. Representative raw data recordings of ICABF, MAP, HR, CO_bpc_, and ETCO_2_ from one subject during the procedure are presented in Figure 1.

**Table 1:** Cardiovascular and respiratory variables during different experimental conditions

	Horizontal		Head‐Down Tilt	
	Rest	HandGrip	Rest	HandGrip
HR (bpm)	58 (52–59)	64^a^ (57–67)	58 (53–60)	67^a^ (58–71)
MAP (mmHg)	72.5 (67.0–76.8)	84.3^a^ (79.9–87.4)	72.2 (68.6–74.4)	85.1^a^ (78.2–87.9)
ICABV (mL)	4.4 (3.4–5.2)	4.1 (3.1–5.1)	4.5 (3.4–5.5)	3.8^a^ (2.7–4.6)
ICABF (mL/min)	260 (188–304)	282 (200–323)	265 (190–287)	257 (182–295)
SV_bpc_ (mL)	87.9 (71.7–98.1)	90.3 (74.5–98.5)	88.6 (75.6– 101.5)	92.5 (77.9–102.2)
CO_bpc_ (L/min)	5.2 (4.0–5.8)	5.9^a^ (4.6–6.6)	5.4^a^ (4.2–6.0)	6.4^a^ (4.8–7.0)
ETCO_2_ (kPa)	4.4 (4.0– 4.6)	4.2 (3.9–4.4)	4.4 (4.1–4.6)	4.2 (4.0–4.4)
RF (Hz)	0.22 (0.19–0.25)	0.26^a^ (0.22–0.29)	0.24 (0.19–0.24)	0.27^a^ (0.22–0.29)
RF (breaths/min)	13 (11–15)	16^a^ (13–17)	14 (11–14)	16^a^ (13–17)

Data from 13 healthy subjects. Medians and 95% confidence intervals calculated by Hodges Lehmann's estimate. ICA, internal carotid artery; BF, blood flow; BV, beat volume; CO_bpc_, cardiac output; SV_bpc_, stroke volume estimates; HR, heart rate; bpm, beats per minute; MAP, mean arterial pressure; ETCO_2_, end‐tidal CO_2_; RF, respiratory frequency. Wilcoxon signed‐rank test was used to test the differences.

a Significance level compared to horizontal rest *P* < 0.01.

**Table 2:** Low‐frequency band (0.05–0.15 Hz) cardiovascular and cerebrovascular variability measured as integrals of wavelet spectral power

	Horizontal		Head‐down tilt	
	Rest	HandGrip	Rest	HandGrip
HR (10^−2^ bpm^2^)	3.6 (1.9–5.1)	5.5 (2.6–7.9)	6.2 (1.3–13.4)	6.1 (3.4–12.8)
MAP (10^−2^ mmHg^2^)	4.8 (2.8–6.0)	6.5^a^ (3.7–8.0)	4.3 (2.8–5.2)	4.3 (2.7–5.9)
ICABV (10^−4^ mL^2^)	7.8 (2.0–10.0)	4.5 (1.8–6.2)	7.0 (3.5–11.6)	6.3 (2.0–13.7)
ICABF (10^−4^ (mL/min)^2^)	19.3 (5.6–26.1)	11.8 (5.4–14.8)	12.5 (7.6–15.5)	17.3 (5.3–28.3)
CO_bpc_ (10^−4^ (L/min)^2^)	2.5 (1.3–3.2)	3.9 (2.2–4.7)	3.5 (1.4–5.4)	4.6^a^ (2.1–5.5)

Data from 12 healthy subjects. Medians and 95% confidence intervals calculated by Hodges Lehmann's estimate.

HR, heart rate; MAP, mean arterial pressure; ICABV, internal carotid artery beat volume; ICABF, internal carotid artery blood flow; CO_bpc_, cardiac output estimates.

a *P* < 0.01 as compared to rest, Wilcoxon signed‐rank test.

**Table 3:** High‐frequency band (0.15–0.4 Hz) cardiovascular and cerebrovascular variability measured as integrals of wavelet spectral power

	Horizontal		Head‐down tilt	
	Rest	HandGrip	Rest	HandGrip
HR (10^−2^ bpm^2^)	17.0 (8.0–25.0)	23.0 (12.0–36.0)	18.0 (9.0–22.0)	28.0 (11.0–56.0)
MAP (10^−2^ mmHg^2^)	4.9 (2.6–6.5)	5.8 (3.1–6.8)	5.3 (2.4–7.0)	5.6 (1.7–7.8)
ICABV (10^−4^ mL^2^)	15.43 (7.5–20.6)	24.9 (6.9–43.1)	23.9 (10.4–28.2)	21.8 (7.8–45.2)
ICABF (10^−4^ (mL/min)^2^)	21.6 (6.3–42.1)	25.6 (10.1–40.5)	38.4 (13.2–48.44)	37.8 (11.3–51.8)
CO_bpc_ (10^−4^ (L/min)^2^)	5.2 (2.9–7.3)	7.9 (2.6–11.2)	6.4 (2.8–16.1)	5.6 (2.5–8.6)

Data from 12 healthy subjects. Medians and 95% confidence intervals calculated by Hodges Lehmann's estimate.

HR, heart rate; MAP, mean arterial pressure; ICABV, internal carotid artery beat volume; ICABF, internal carotid artery blood flow; CO_bpc_, cardiac output estimates.

**Table 4:** Phase angle, coherence and phase synchronization index *γ* for pairs of cardiovascular variables at the lower frequency interval, at Mayer wave frequency and at individual subjects’ peak respiratory frequency

	Horizontal		Head‐down tilt	
	Rest	HandGrip	Rest	HandGrip
Lower frequency interval (0.05–0.15 Hz)				
MAP–ICABF	0.23	0.27	0.19	0.22
Median *γ*	(0.12–0.28)	(0.17–0.36)	(0.12–0.25)	(0.10–0.30)
CO_bpc_–ICABF	0.22	0.29	0.14	0.16
Median *γ*	(0.15–0.28)	(0.19–0.34)	(0.08–0.17)	(0.10–0.23)
Mayer wave frequency				
MAP–ICABF				
Phase (rad)	0.5 (0, 0.9)	1.0 (0.5, 1.6)	0.5 (−0.2, 1.3)	1.3 (0.7, 1.9)
Coherence	0.53 (0.4–0.59)	0.66 (0.5–0.69)	0.56 (0.4–0.65)	0.50 (0.32–0.59)
*γ*	0.62 (0.43–0.7)	0.68 (0.56–0.73)	0.54 (0.33–0.65)	0.52 (0.25–0.56)
CO_bpc_–ICABF				
Phase (rad)	−0.7 (−1.1, −0.2)	0.0 (−0.5, 0.4)	−0.8 (−1.5, −0.1)	0.1 (−0.6, 0.8)
Coherence	0.61 (0.51–0.65)	0.59 (0.42–0.66)	0.46 (0.29–0.57)	0.46 (0.3–0.54)
*γ*	0.56 (0.42–0.63)	0.53 (0.4–0.63)	0.47 (0.33–0.56)	0.5 (0.32–0.57)
HR–ICABV				
Phase (rad)	−2.6 (−2.8, −2.3)	−2.7 (−3.1, −2.3)	−2.8 (−3.4, −2.2)	−3.0 (−3.4, −2.6)
Coherence	0.78 (0.64–0.83)	0.76 (0.65–0.82)	0.71 (0.64–0.75)	0.68 (0.61–0.73)
*γ*	0.62 (0.43–0.7)	0.63 (0.47–0.71)	0.52 (0.4–0.58)	0.47 (0.38–0.53)
Peak respiratory frequency				
MAP–ICABF				
Phase (rad)	0.1 (−0.3, 0.5)	−0.1 (−0.5, 0.3)	−0.3 (−0.9, 0.4)	−0.4 (−1.3, 0.3)
Coherence	0.64 (0.53–0.72	0.53 (0.38–0.58)	0.58 (0.41–0.65)	0.63 (0.47–0.72)
*γ*	0.51 (0.38–0.59)	0.34 (0.24–0.4)	0.34 (0.27–0.49)	0.51 (0.33–0.6)
CO_bpc_–ICABF	–	–	–	–
Phase (rad)	0.52 (0.37–0.59)	0.47 (0.30–0.52)	0.53 (0.39–0.64)	0.49 (0.40–0.55)
Coherence	0.35 (0.18–0.46)	0.30 (0.18–0.36)	0.42 (0.23–0.48)	0.40 (0.23–0.49)
*γ*				
HR–ICABV				
Phase (rad)	−3.1 (−3.3, −2.8)	−3.0 (−3.2, −2.9)	−3.1 (−3.3, −2.8)	−3.0 (−3.2, −2.9)
Coherence	0.92 (0.85– 0.94)	0.9 (0.78–0.95)	0.95 (0.83– 0.96)	0.89 (0.79–0.92)
*γ*	0.85 (0.74–0.88)	0.83 (0.66–0.89)	0.89 (0.71–0.93)	0.79 (0.66–0.85)

Data from 12 healthy subjects. Values for coherence and phase synchronization index *γ* are medians and 95% confidence intervals calculated by Hodges‐Lehmann estimates. Values for phases are the circular means (–*π*,* π*) with 95% CI.

HDT, head‐down tilt; HG, handgrip; MAP, mean arterial pressure; ICABF, internal carotid artery blood flow; CO_bpc_, cardiac output estimates; HR, heart rate; ICABV, internal carotid artery beat volume.

The HR–ICABV pair showed high coherence and *γ* at the respiratory frequency, in all experimental states (Table 4). These variables fluctuated in inverse phase, with increases in HR to coincide with decreases in ICABV and vica versa.

### Steady state cardiovascular changes induced by isometric handgrip and head‐down tilt

Isometric handgrip resulted in an increase in MAP (+16%, *P* < 0.001), CO_bpc_ (+15%, *P* < 0.001) and HR (+10%, *P* < 0.001, Table 1). SV_bpc_ did not change. ICABV volume tended to decrease (*P* = 0.08) but ICABF was preserved from rest to HG (Table 1).

The slight HDT by −10°did not affect MAP, HR, SV_bpc_, ICABV, and ICABF, though CO_bpc_ increased slightly (+4%, *P* = 0.001, Table 1).

CO_bpc_ and MAP increased significantly from rest to HDT + HG (CO_bpc_: +23%, (*P* < 0.001), MAP: +12%, (*P* = 0.007, Table 1)). HR also increased by 16%, (*P* < 0.001). On the contrary, ICABV decreased by 14% (*P* = 0.002) and ICABF was unchanged from rest to HDT + HG due to the increase in HR.

ETCO_2_ did not differ between experimental states (Table 1). The Friedman test for three related samples revealed no change in ETCO_2_ between the first, the second and the third minute of the HG maneuver, neither in the horizontal supine position (*P* = 0.4) nor during HDT (*P* = 0.8).

### Phase synchronization at rest

#### Low‐frequency interval at rest

**Figure 2:**
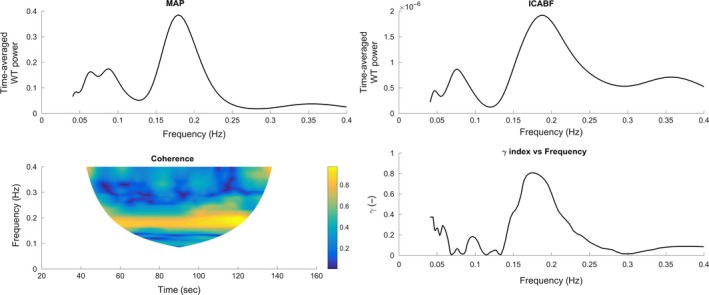
Time averaged wavelet power for mean arterial pressure (MAP) and internal carotid artery blood flow (ICABF), contour plot of coherence and plot of *γ* index against frequency during rest in one subject. Two peaks are identified in the power spectrums of both variables and the *γ* index: the Mayer wave peak at around 0.1 Hz and the respiratory peak (at ~0.2 Hz in this subject).

**Figure 3:**
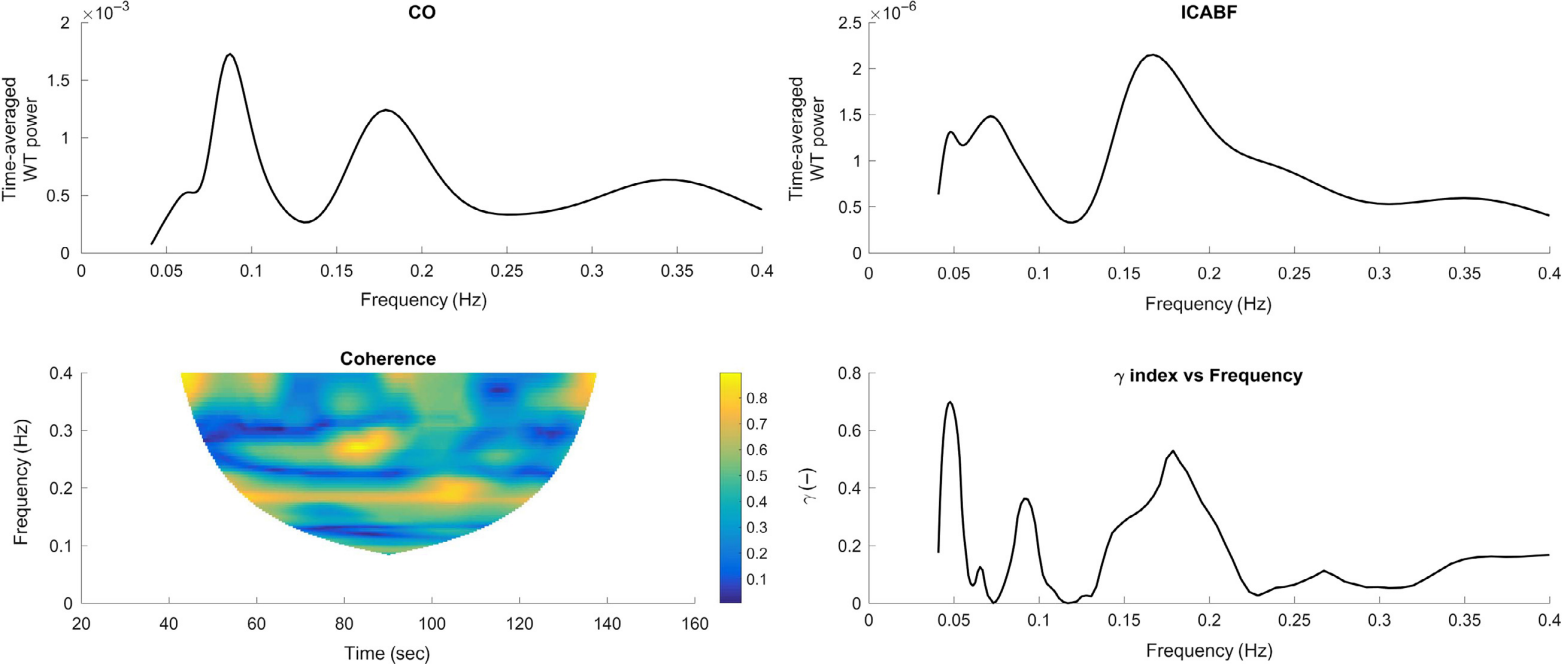
Time averaged wavelet power for cardiac output (CO_bpc_) and internal carotid artery blood flow (ICABF), contour plot of coherence and plot of *γ* index against frequency during rest in one subject. Two peaks are identified in the power spectrums of both variables and the *γ* index: the Mayer wave peak at around 0.1 Hz and the respiratory peak (at ~0.2 Hz in this subject).

For the MAP–ICABF pair, median *γ* over the entire LF interval was low (Table 4) at rest, indicating a high phase difference variability between MAP and ICABF and thus a functioning dynamic CA (Latka et al. 2005). For MAP, a peak in the wavelet power spectrum at 0.08–0.12 Hz was present in all subjects at rest, representing Mayer waves (Fig. 2). Similar peaks around 0.1 Hz were observed also in the wavelet power spectra of CO_bpc_ (Fig. 3) and HR. Coherence and synchronization index *γ* between the selected variable pairs were higher at the Mayer wave peak than when measured as median of the entire LF interval (Fig. 2).

A *γ* of comparable magnitude to the MAP–ICABF pair was observed for the variable pair CO_bpc_–ICABF during rest (Table 4). Again, a peak in *γ* was observed around 0.1 Hz. High coherence and *γ* was also observed for the variable pair HR–ICABV; the oscillations of the two signals being in inverse phase at the Mayer wave peak.

#### High‐frequency interval at rest

In the HF interval, a peak in the power spectrum related to the subjects’ respiration (varying between 0.18 and 0.34 Hz) was observed for HR, MAP, CO_bpc_, and ICABF. Moderate values of coherence and *γ* at the respiratory frequency were observed for the variable pair MAP–ICABF (Table 4). A positive mean phase difference (0.1 rad) between MAP and ICABF indicated that the former “drove” the latter and that the fluctuations of the signals were in phase. The circular mean phase difference for the CO_bpc_–ICABF pair was not possible to calculate in the HF interval, as individual values of phase were equally spread around the unit circle. Low values of *γ* were observed for the CO_bpc_–ICABF pair at the respiratory frequency.

### Phase synchronization during horizontal handgrip

**Figure 4:**
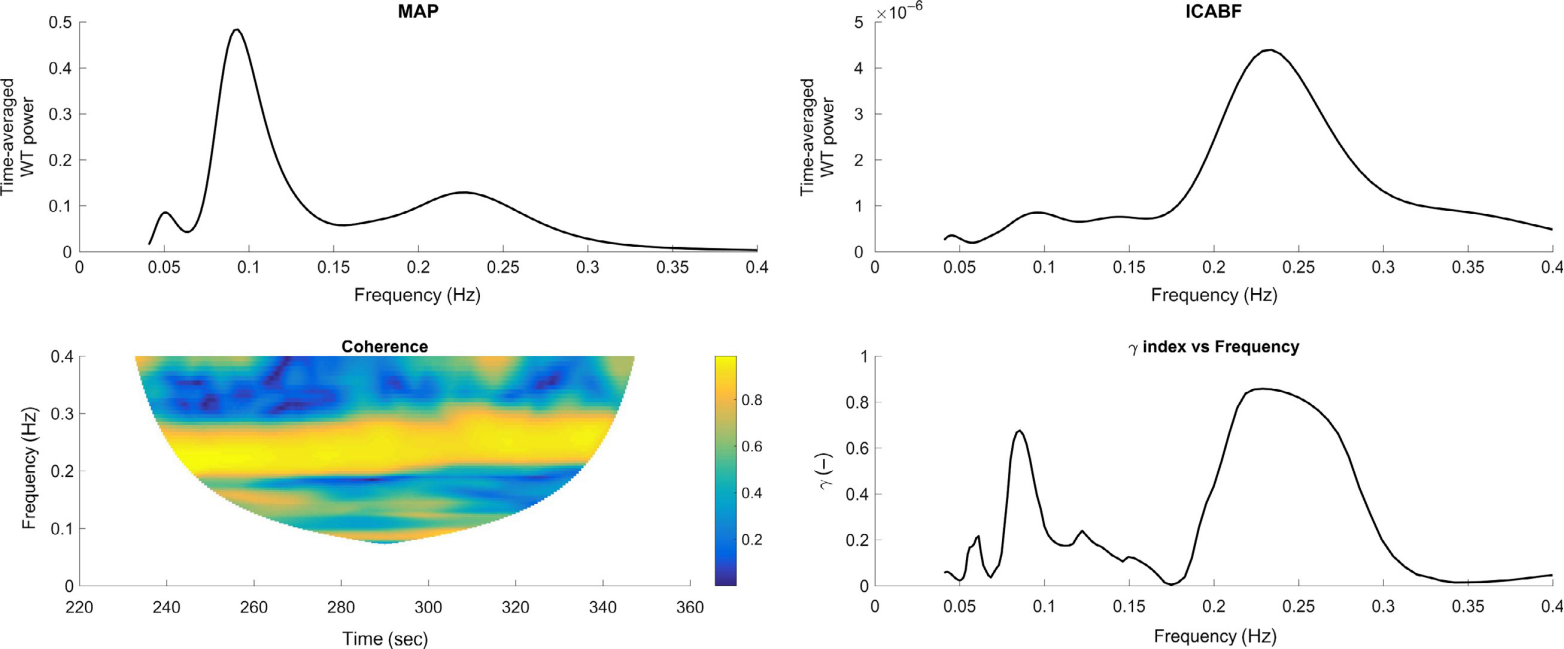
Time averaged wavelet power for mean arterial pressure (MAP) and internal carotid artery blood flow (ICABF), contour plot of coherence and plot of *γ* index against frequency during handgrip in the horizontal position in one subject (same as in Fig. 2). Two peaks are identified in the power spectrums of both variables and the *γ* index: the Mayer wave peak at around 0.1 Hz and the respiratory peak (at ~0.25 Hz). Compared to rest, a higher Mayer wave peak for MAP and a higher *γ* index at 0.1 Hz are observed.

**Figure 5:**
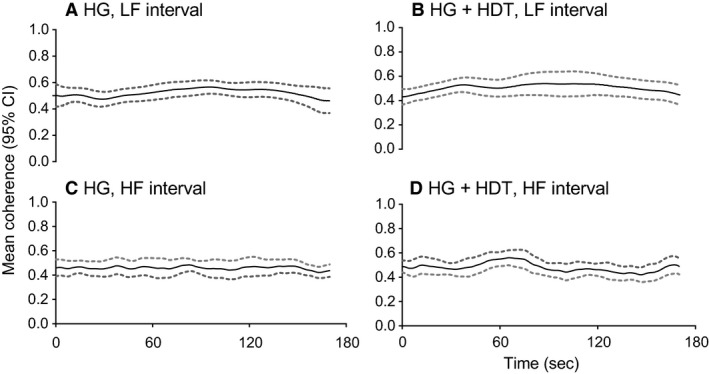
Group mean (black line) and 95% CI (grey dashed lines) of frequency‐averaged wavelet coherence between mean arterial blood pressure (MAP) and internal carotid artery blood flow (ICABF) plotted over time, during handgrip (HG) and head‐down tilt combined with HG (HDT + HDT), for the low frequency (LF) and the high frequency (HF) interval. *N* = 12. The frequency‐averaged coherence between MAP and ICABF did not change over time during the HG maneuver in either body position or frequency interval.

Figure 4 shows the time‐averaged power spectrums of MAP and ICABF, the contour plot of their coherence, and the synchronization index *γ* plotted against frequency (LF; 0.05–0.4 Hz) for the MAP–ICABF pair during HG. The Mayer wave peak was clearly amplified during isometric exercise. Phase variability with low synchronization (low *γ* values) was observed at low frequencies. At higher frequencies, and particularly around RF, coherence increased and high values of *γ* was observed (Fig. 4). The frequency‐averaged coherence for the MAP–ICABF variable pair did not change over time during HG in either frequency interval (Fig. 5A and C).

#### Low‐frequency interval

For the MAP–ICABF and CO_bpc_–ICABF pairs, median *γ* over the entire LF interval did not change from rest to isometric handgrip (Table 4), and similar to during rest the synchronization index *γ* showed a peak at 0.1 Hz. High values of *γ* were observed for the HR–ICABV pair at 0.1 Hz, their signals in inverse phase similar to during rest.

#### High‐frequency interval

For the MAP–ICABF pair, the synchronization index *γ* at the RF tended to be lower during HG than at rest (*P* = 0.03), and significantly lower at the RF than at the Mayer wave peak (*P* = 0.007). For the CO_bpc_–ICABF pair coherence and *γ* at the RF were moderate (Table 4), indicating that respiration‐induced changes in CO_bpc_ contributed to ICABF variability during HG. Individual phase angles between CO_bpc_ and ICABF were spread around the unit circle (high between‐subject variability).

### Phase synchronization during head‐down tilt

#### Low‐frequency interval

For the MAP–ICABF and CO_bpc_–ICABF pairs, median *γ* over the entire LF interval were low and did not change from rest to HDT (Table 4). The Mayer wave peak in synchronization was present. For the HR–ICABV pair, relatively high values of *γ* were observed; the signals remaining in inverse phase, similar to at rest.

#### High‐frequency interval

The synchronization index *γ* for the MAP–ICABF pair at the RF decreased (*P* = 0.007, Table 4), while the *γ* for the CO_bpc_–ICABF pair at the RF remained low and unchanged from rest.

### Phase synchronization during head‐down tilt and handgrip

#### Low‐frequency interval

For the MAP–ICABF variable pair, median *γ* across the entire LF interval did not change from rest (see above) to HDT + HG (0.22, 0.10–0.30), but again phase synchronization index *γ* was higher at 0.1 Hz. The phase angle between MAP and ICABF at 0.1 Hz was significantly increased (*P* = 0.01) during HDT‐HG compared to rest.

For the CO_bpc_–ICABF variable pair, median *γ* across the entire LF interval was low (0.16, 0.1–0.23) during HDT‐HG, but also for this pair *γ* was increased at 0.1 Hz. HR and ICABV remained in inverse phase, as at rest.

#### High‐frequency interval

In the HF interval, cardiovascular and cerebrovascular variability did not change during HDT‐HG compared to rest. The synchronization index *γ* at the RF also remained unchanged for all studied variable pairs (MAP–ICABF, CO_bpc_–ICABF, HR–ICABV).

The frequency‐averaged coherence for the MAP–ICABF variable pair did not change over time during HDT + HG in either frequency interval (Fig. 5B and D).

## Discussion

This study examined the effect of central cardiovascular variability on cerebrovascular variability during rest, isometric exercise, HDT, and their combination. Aside from the well‐investigated relationship between arterial blood pressure and CBF, CO_bpc_, and HR were investigated as possible sources of cerebrovascular variability. To this end we employed wavelet analysis, as it detects events both in time and frequency and addresses the nonlinearities of cardiovascular oscillations. Our main findings indicate that (1) Overall, there was a low degree of synchronization between slow oscillations in ICABF and slow oscillations in MAP during rest, isometric HG, HDT, and HDT + HG, and steady state ICABF did not change. This indicates an intact dynamic CA, which was unaffected by the increases in MAP and sympathetic activity during isometric HG. The oscillations were more synchronized at the Mayer wave frequency, that is around 0.1 Hz. (2) Variabililty in cerebrovascular flow was independent of the slow spontaneous oscillations in CO_bpc_, during all experimental states, as indicated by the low degree of synchronization between oscillations in ICABF and CO_bpc_. Faster CO_bpc_ variations, that is., at the respiratory frequency, contributed moderately to ICABF variability. (3) HR oscillations remained synchronized and in inverse phase with ICABV oscillations, both in the LF and in the HF interval in all experimental states. These findings expand on previous findings from our group (Skytioti et al. 2017).

Steady‐state results showed that despite significant changes in MAP and CO_bpc_ during HG, neither in the horizontal supine position nor during HDT did median ICABF change from rest. Thus, regulatory mechanisms must have counteracted the increase in arterial blood pressure and CO_bpc_, maintaining CBF at baseline levels.

The dynamic CA in this study was unaffected by the autonomic and cardiovascular changes elicited by exercise and HDT, as reflected by the absence of change in CBF. Whether CO changes may induce changes in CBF independently of MAP is a debated issue. Deegan et al. reported no change in CBF following an increase in CO after a thigh cuff release maneuver. In contrast, Ogoh et al. reported a linear relationship between CO (manipulated by lower body negative pressure and albumin infusion) and middle cerebral artery flow velocity (Ogoh et al. 2005). We have also found a linear relationship between simultaneous Doppler ultrasound recordings of CO and ICABF during normovolemia and hypovolemia induced by lower body negative pressure, despite no change in MAP (Skytioti et al. 2016). In the present study, ICABF did not change despite a 23% increase in CO_bpc_ and a 12% increase in MAP during HDT + HG. Cerebral vasoconstriction due to sympathetic stimulation during isometric handgrip might have counteracted the increase in CO_bpc_ and MAP during HDT + HG (Ogoh et al. 2005). The different methods employed for the manipulation of CO (thigh cuff release, lower body negative pressure, albumin infusion, isometric exercise) induce different autonomic responces, which may be the reason for the diversity in the literature concerning the role of CO in the regulation of CBF.

HG maneuver alone increased variability, whereas HDT + HG did not alter MAP variability in the LF range (Table 2). Human and animal studies have shown that arterial blood pressure variability in the LF range is related to the sympathetic activity (Pagani et al. 1986; Hilz et al. 2013). Our results indicate an increase in the sympathetic activity during HG alone compared to rest (increased MAP variability, Table 2). The lack of increase in LF MAP variability during HDT + HG probably resulted from baroreceptor loading and sympathetic withdrawal (Cooke et al. 2003) caused by the HDT.

### Cerebral autoregulation – regulation against changes in pressure

In the LF interval low phase synchronization index between ICABF and MAP suggested an operative dynamic CA. Lower values of the synchronization index were observed below 0.15 Hz. This finding is in line with existing literature concerning CA effectivity at low frequencies (Zhang et al. 1998; Brys et al. 2003; Panerai 2008; Tan 2012; Fraser et al. 2013; Tan and Taylor 2014; Claassen et al. 2016). The median synchronization index for MAP–ICABF across the LF interval (0.05–0.15 Hz) did not change from rest to either HG, HDT or their combination. The frequency‐averaged coherence for MAP–ICABF was also unchanged over time during HG and HDT + HG (Fig. 5A and B). These findings indicate that CA was unaffected by the sympathetic stimulation during isometric exercise, both in the horizontal position and during HDT.

Variations in CBF and systemic blood flow in the HF interval are mainly related to respiration (Cohen and Taylor 2002; Rowley et al. 2007). A moderate degree of phase synchronization was observed at the RF for the MAP–ICABF variable pair at rest, in line with existing literature (Peng et al. 2010). Synchronization between MAP and ICABF at the respiratory frequency tended to decrease from rest to HG (Table 4) and the frequency‐averaged coherence for MAP–ICABF did not change over time during the HG maneuver (Fig. 5C and D). A sympathetic cerebral vasoconstriction has been described during exercise, protecting the brain from elevations in cerebral perfusion pressure (Ogoh and Ainslie 2009). Sympathetic cerebral vasoconstriction due to isometric exercise might have been responsible for the loss of synchronization in the MAP–ICABF variable pair observed in our experiments during HG.

### Regulation against changes in CO_bpc_

ICABF in this study did not change despite elevations in CO_bpc_ during HG, HDT and HDT + HG and no change in ETCO_2_. This finding is in agreement with a study by Deegan et al. (2010) in which elevations in the cardiac index of healthy volunteers had no effect on middle cerebral artery blood flow velocity. Our group has however previously found a linear relationship between CO and ICABF during central hypovolemia (Skytioti et al. 2016). The response of ICABF to CO changes might therefore be differential; cerebrovasculature may counteract the elevations but not completely the reductions in CO. Cerebral vasoconstriction due to sympathetic activation during HG may prevent elevations in ICABF, while the same mechanism may contribute to ICABF reductions during hypovolemia.

In agreement with the steady state data, a low median *γ* was found for the CO_bpc_–ICABF variable pair in the LF interval during rest, suggesting that slow spontaneous CO_bpc_ oscillations were counterregulated and not transferred to the cerebral circulation. Cerebral autoregulatory mechanisms may thus buffer slow spontaneous oscillations in flow in addition to perfusion pressure. HG, HDT, and HDT + HG did not affect the relationship between CO_bpc_ and ICABF in the LF interval.

At 0.1 Hz, moderate *γ* values and phase shifts close to zero were observed between oscillations in CO_bpc_ and ICABF. This increased synchronization at the Mayer wave frequency may be explained by sympathetically induced variability in both CO_bpc_ and ICABF.

### The buffering effect of heart rate variability

High HR variability was observed at 0.1 Hz, and these HR oscillations were synchronized and in inverse phase with ICABV oscillations. This was likely due to the baroreflex response to Mayer waves in MAP and cardiac stroke volume (Hilz et al. 2013).

Confirming previous findings (Skytioti et al. 2017), the respiratory‐related HR variations and ICABV variations were in inverse phase and with a very high synchronization index. Fast the Fourier Transform algorithm and cross‐spectral analysis were employed in this previous study to examine the relationship between respiration‐induced variations in HR and ICABV; we found that HR variations and ICABV variations were in inverse phase and highly coherent while ICABF showed little variability in spontaneously breathing healthy individuals. In this study, the close relationship between oscillations in HR and ICABV did not change with HG, HDT or their combination. We suggest that respiratory sinus arrhythmia may play a role as a regulatory mechanism which buffers ICABV fluctuations and stabilizes ICABF over the respiratory cycle during rest. This study indicates that this mechanism is operative also during static exercise. Increased respiratory sinus arrhythmia was observed during a −45° HDT in one case report (Baden et al. 2014); we did not find an increased HR variability during −10° HDT.

In a recent study, Marmarelis et al. (2016) assessed the contribution of HR changes to CBF velocity changes by inserting HR as a third input in a two‐input (arterial blood pressure and ETCO_2_) dynamic nonlinear model. Their results imply that HR variability may contribute to CBF velocity variability, independently of changes in arterial blood pressure and ETCO_2_. This finding is in line with previous (Skytioti et al. 2017) and present results from our group demonstrating a role of HR variability in the regulation of CBF.

### Phase synchronization peaks

Two frequency peaks were identified for the phase synchronization index of the examined variable pairs; one at 0.1 Hz (Mayer waves) and one at the subjects’ respiratory frequency. This finding is in agreement with previous studies using the synchronization index *γ* (Latka et al. 2005; Peng et al. 2010). Mayer waves and respiration‐related waves have been identified also in the intracranial cerebrospinal fluid flow (Strik et al. 2002), supporting the existence of oscillations in the cerebral circulation.

### The Mayer wave peak

Arterial blood pressure oscillations around 0.1 Hz (Mayer waves) are considered to represent reflex‐mediated changes in sympathetic outflow to systemic vasculature (Cohen and Taylor 2002). There is also evidence that sympathetic activity directly induces or modulates oscillations in CBF velocity at 0.1 Hz (Hilz et al. 2013). Our results are in line with these findings.

### The respiration peak

In the HF interval (0.15–0.4 Hz), respiration is the main source of cardiovascular variability (Toska and Eriksen 1993; Kuo et al. 1998). We found moderate synchrony between respiratory‐related variations in MAP and CO_bpc_ respiratory‐related variations in ICABF (Table 4), indicating a mechanism for the respiratory variability in CBF. In the HF interval, oscillations in MAP and CBF have been shown to derive from mechanical oscillation in venous return and CO (Toska and Eriksen 1993; Elstad et al. 2001; Hilz et al. 2013).

### Static versus dynamic cerebral autoregulation

For practical and clinical reasons it is not possible to construct the full Lassen's curve for one patient, as it would require the patient to be exposed to a wide range of arterial blood pressures. It is not usually feasible to induce hemodynamic changes (thigh‐cuff release, lower body negative pressure, pharmacologically induced) to a patient in order to test CA. It is however possible to continuously monitor dynamic CA using metrics derived from the analysis of low‐frequency spontaneous fluctuations in arterial pressure (albeit with a low magnitude) and CBF (Latka et al. 2005; Panerai 2008; Czosnyka et al. 2009; Peng et al. 2010). It is therefore essential that the metrics of dynamic CA reflect the ability of the cerebrovasculature to respond to changes in pressure and flow. The relationship between static and dynamic cerebral autoregulation has been previously assessed. A strong correlation was found between static and dynamic cerebral autoregulation in young healthy patients under general anesthesia with propofol (intact CA) and isoflurane (impaired CA, same patients)(Tiecks et al. 1995). In contrast, an absence of linear relationship between metrics of static and dynamic cerebral autoregulation has been reported in aged adults (de Jong et al. 2017). The different populations and methodologies and the drugs used in these studies are possible explanations for this discrepancy. The young healthy adults of the first study had intact CA during propofol anesthesia and subsequently demonstrated a uniform CA impairment during isoflurane anesthesia. In the second study, the autoregulatory capacity varied in the aged population examined from intact to impaired. Taking into consideration these studies we could speculate that dynamic CA metrics reflect static CA during conditions known to impair CA such as isoflurane anesthesia (Tiecks et al. 1995) or traumatic brain injury (Czosnyka & Miller 2014). In the healthy population however dynamic and static CA metrics may be not correlated due to the large inter‐individual variability. It is also possible that different mechanisms control cerebrovascular resistance and the CBF response to arterial blood pressure changes over different time scales (de Jong et al. 2017).

### Clinical implications

Impaired cerebral autoregulation has been reported in several pathological conditions (Donnelly et al. 2015). In traumatic brain injury (Depreitere et al. 2014) and acute ischemic stroke (Immink et al. 2005) poor cerebral autoregulatory capacitance is related to poor outcome. A major obstacle for any monitoring system of cerebral autoregulation is that this mechanism is likely to be locally disrupted, specifically in traumatized or ischemic brain areas. Therapy directed to improve CBF may therefore result in “steal effects” where improved overall flow is unproportionally directed to healthy areas of the brain.

The adequacy of CBF is not routinely monitored in a clinical setting where the patient cannot cooperate with neurological assessment, for example, due to sedation, anesthesia, or critical illness, although several studies have reported the usefulness of CBF measurements in these patient groups (Kalmar et al. 2012; Kvandal et al. 2013; Schramm et al. 2014). For everyday monitoring of patients in general anesthesia or during critical care, clinicians therefore usually rely on relatively crude and indirect measures, such as arterial blood pressure or cerebral perfusion pressure (MAP – intracranial pressure) as indicators of blood supply to the brain. A continuous monitoring of aspects of cerebral autoregulation would be a powerful tool that conceivably could help guiding clinical decisions and therapy.

CBF responses to changes in CO is also of clinical interest, as situations that result in reduced CO also may reduce CBF, in spite of a preserved MAP (Ogoh et al. 2005; Skytioti et al. 2016). Therefore, preserving cerebral perfusion pressure between the upper and the lower limit of Lassen's traditional cerebral autoregulation curve does not guarantee adequate cerebral perfusion. Bedside continuous monitoring of the dynamic interrelationship between CO and CBF in various disease states could contribute to a better understanding, and ultimately management, of CBF regulation.

### Methodological considerations and limitations

A methodological concern in this study was whether the diameter of the ICA, which was measured once in the beginning of each experiment, remained stable throughout the experiment or changed in response to HDT and HG. We have not found previous studies that examined ICA diameter during mild HDT of −10°. However, Montero and Rauber examined ICA diameter changes during sustained (30 min) HDT of ‐30 degrees, and during the initial 10 min of their experiments no change in ICA diameter was reported (Montero and Rauber 2016). In our experiments, subjects were positioned in −10° HDT for 6 min. Rubenfire et al. (2002) found no detectable change in common carotid artery diameter during 120 sec of isometric handgrip of 33% of maximal voluntary contraction. It is likely that larger changes in MAP may be required to change the ICA diameter. According to Lewis et al. (2015) a 20% reduction in MAP was accompanied by a decrease in 5% in ICA diameter. Therefore we assumed that ICA diameter did not change during HDT and/or HG.

The use of the synchronization index as a measure of CA has not been validated as much as parameters derived from transfer function analysis or other measures (autoregulatory index). However, wavelet analysis has been extensively used to evaluate dynamic CA (Kvandal et al. 2013; Addison 2015; Gao et al. 2015; Tian et al. 2016). In addition, wavelet phase synchronization analysis among other methods has been proposed to address the nonstationarity that characterizes dynamic CA (Panerai 2014).

SV and CO was not measured directly in the present study; the Finometer calculated estimates of these variables from the arterial blood pressure curve using the Modelflow algorithm (Bogert and van Lieshout 2005). Our group has previously reported a close correlation between Modelflow SV estimates and SV measured by Doppler ultrasound despite small differences in the absolute values (Holme et al. 2016).

The probe used to measure velocity in ICA was handheld by a trained operator. The operator of a handheld probe may introduce an error into the angle of insonation during velocity recordings. A deviation of 5° yields an error of 10% in ICA blood velocity (Yamamoto et al. 2006).

ETCO_2_ was necessarily calculated once per respiratory cycle (end‐expiration). Mitsis et al. showed that ETCO_2_ variations have a considerable effect on CBF at frequencies below 0.04 Hz (Mitsis et al. 2004). In order to evaluate a possible effect of ETCO_2_ variations on ICABF in our study, we calculated the median *γ* index for the system ETCO_2_‐ICABF in the LF and HF interval, which was found to be very low (below 0.1). Taking into account the previous reports, the fact that ETCO_2_ did not change during HG, and the low *γ* index in our study, we assumed that ETCO_2_ fluctuations did not affect cerebrovascular variability.

## Conclusion

Overall ICABF did not change during static handgrip, head‐down tilt, or their combination, despite significant changes in CO_bpc_ and MAP. Dynamic cerebral autoregulation was intact at baseline in all the subjects as indicated by the low synchronization index between MAP and ICABF at frequencies below 0.15 Hz. Wavelet analysis revealed a low degree of synchronization between oscillations in cerebral blood flow and MAP over the 0.05–0.15 Hz interval during HG, HDT and HDT + HG; this was interpreted as an operative dynamic cerebral autoregulation. CBF also seemed to be regulated against slow CO_bpc_ oscillations, as indicated by low synchronization between CO_bpc_ and ICABF variability. The high synchronization and the inverse phase between oscillations in HR and ICABV indicated that HR variations may act as a regulatory mechanism, minimizing CBF variability both in the LF and HF interval.

## Conflict of Interest

No conflict of interest declared.
